# Phase equilibria and drug partitioning ability of betaine based aqueous two-phase systems

**DOI:** 10.1038/s41598-025-88326-4

**Published:** 2025-02-13

**Authors:** Mohammed Taghi Zafarani-Moattar, Hemayat Shekaari, Soheila Asadollahi

**Affiliations:** Department of Physical Chemistry, University of Tabriz, Tabriz, Iran

**Keywords:** Aqueous two-phase systems, Betaine, PEGDME_250_, Potassium salt, Drug partitioning, Thermodynamics, Green chemistry

## Abstract

The aqueous two-phase system (ATPS) as an environmentally friendly technique has a high potential for extraction of drugs, proteins, metals, etc. In this work first the formation of ATPSs by betaine, which is a novel, low toxic, and high biodegradable material, and polyethylene glycol or phosphate salts (K_3_PO_4_ and K_2_HPO_4_) were investigated temperatures of 298.15, 308.15, 318.15 K and atmospheric pressure (≈ 85 kPa). The effect of increasing temperature on the binodal curves was investigated. The experimental binodal data were correlated using two empirical nonlinear three parameter expressions developed by Merchuk, and Zafarani-Moattar et al. and the two-parameter modified equation derived from effective excluded volume theory. Then, the experimental tie-line data were represented with Othmer-Tobias and Setschenow correlations. Finally, in these ATPSs the partition behavior of analgesic drugs, namely acetaminophen, salicylic acid, and ceftriaxone were investigated by measuring of partition coefficient and extraction efficiency. The trend of obtained results showed that the selected drugs were successfully extracted into the top phase of studied ATPSs with a partition coefficient greater than one and in the salt systems extraction efficiency was obtained up to 98% and in the case of polymer systems was obtained up to 8% at different tie-lines and it was found that the hydrophobicity of partitioned drugs is responsible for this phenomenon.

**Supplementary Information:**

The online version contains supplementary material available at 10.1038/s41598-025-88326-4.

## Introduction

The thermodynamic studies of aqueous two-phase systems (ATPSs) are important since they can be used for the separation and extraction of the different solutes such as drugs and phenolic compounds^1–4^. The use of ATPSs are highly attractive compared to other methods because they are eco-friendly, low cost, capable of continues operation, scaling-up possibility, low energy requirement and is widely applied, especially for the concentration and purification of biomolecules^5–7^. Water as the rich reactive substrate in the top and bottom phases in ATPS forms a gentle environment for biomolecules to separate. Different types of ATPSs containing two polymers, polymer-salt, alcohol -salt, polymer-ionic liquid and salt-ionic liquid have been investigated^8–10^along with their applications for extraction and separation of different solutes. In recent years, in order to reduce the use of harmful substances and environmental pollution, researchers focused on the new and green materials for making suitable ATPSs. For this purpose, choline chloride^11,12^and betaine^13^, which have been prepared through simple and green precursors, have been used as a component of ATPS, recently.

The information regarding the phase behavior and extraction performance of ATPSs containing betaine is scarce. The phase separation of aqueous betaine solutions is also influenced by the salts, the effect depending on both their concentration and type. Often, a sufficiently concentration of salt in a single betaine-water system can cause phase separation to yield a two phase system with salt-rich, betaine-poor bottom phase and salt-poor, betaine-rich top phase^14^. The relative effectiveness of various salts in improvement phase separation is determined by the Hofmeister series, which is a classification of ions based on their salting-out ability. The contribution of the anion is more important than that of the cation in determining the effectiveness of a salt. The multivalent anions like HPO_4_^2–^ and SO_4_^2– ^are most effective in inducing phase separation with betaine^15^.

Betaine is very promising material due to its plant origin and unique properties. Betaine (2-(trimethyl azaniumyl) acetate) is an organic nitrogenous and environmentally safe compound, found for the first time in sugar beet juice (Beta vulgaris)^13,16^. In addition to the mentioned properties, betaine is stable, nontoxic and highly soluble in water^17–19^. The results obtained from previous studies have shown that both choline chloride and betaine have the ability to form a two-phase system with phosphate salts, but choline chloride requires more mineral salts than betaine to form two phases. Also, due to less methyl groups which could enhance hydrogen bonding with water, thus competed with the inorganic salt ions for the formation of hydration complexes, betaine forms a more stable two-phase system than choline chloride^20–22^. Recently, phase behavior and drug extraction ability of ATPSs containing betaine and polyethylene glycol or potassium salts for protein extraction have been studied without giving the corresponding binodal or tie-line data^20^. In our previous work from measurement of water activity and volumetric properties of aqueous betaine solutions containing polyethylene glycol di-methyl ether) with molar mass 250 g.mol^−1^ (PEGDME_250_) or phosphate salts we concluded that these ternary solutions could make ATPSs^23^. Further studies are required regarding the ability of these systems to form ATPS in some detail and their efficient use in extraction processes.

In this work, to obtain phase behavior of ATPSs containing betaine in some detail first the ability of betaine to form two phase system in presence of a PEGDME_250_ or phosphate salts have been studied. The binodal and tie-line data were measured and reported for the {betaine + PEGDME_250_ + H_2_O}, {betaine + K_3_PO_4_ + H_2_O} and {betaine + K_2_HPO_4_ + H_2_O} systems at *T*= (298.15, 308.15 and 318.15) K and atmospheric pressure (≈ 85 kPa). Then, the extraction performances of the mentioned ATPSs have been investigated for analgesic drugs, namely acetaminophen, salicylic acid and ceftriaxone. The obtained experimental binodal data were correlated using empirical Merchuk^15^, Zafarani-Moattar and Nemati^24^equations and the modified effective excluded volume (EEV) model^25^. Also, performances of the Othmer-Tobias, Bancraft^26,27^and Seteschenow^28^ equations were examined for reproducing the experimental tie-lines compositions.

## Experimental section

### Materials

The necessary information corresponding to the materials used in this research have been given in Table 1. The poly ethylene glycol di-methyl ether (CAS registry No. 24991-55-7) with a molar mass of 250 g.mol^−1^(PEGDME_250_), tri-potassium phosphate and di-potassium hydrogen phosphate were supplied by Merck. Betaine with a minimum purity of 98% was obtained from Jinan Grace Industry. Acetaminophen and ceftriaxone (> 99.5 wt %) were obtained from Zahravi pharmaceutical company (Iran). Salicylic acid, ethylene dichloride (1, 2-dichloroethane) and sulfuric acid were also provided by Merck. All materials were used without further purification and double distilled and deionized water was used in preparation of solutions. Water content of the chemicals were measured by the Karl-Fischer method as shown in Table 1. The maximum uncertainty in determining the mass fraction of polymer and salt was found to be 0.002.

**Table 1:** A brief summary of the purity and specifications of the used materials.

Chemical name	CAS Reg. No.	Source	Purity, mass fraction	Molecular formula	^a^ Water contents
Poly ethylene glycol di- methyl ether 250	24,991-55-7	Merck	> 0.995	CH_3_O(CH_2_CH_2_O)_n_CH_3_	0.0003
Betaine	107-43-7	Jinan Grace Industry	> 0.98	C_5_H_11_NO_2_	0.0008
tri-potassium phosphate	57-50-1	Merck	> 0.995	K_3_PO_4_	0.0009
di-potassium hydrogen phosphate	7647-14-5	Merck	> 0.995	K_2_HPO_4_	0.0003
Acetaminophen Salicylic acid Ceftriaxone Ethylene Dichloride Sulfuric acid	103-90-2 69–72-7 104376-79-6 107-06-2 7664-93-9	Zahravi CO. Merck Zahravi CO. Merck Merck	> 0.995 > 0.995 > 0.995 0.96–0.98 ≥ 0.995	C_8_H_9_NO_2_ C_7_H_6_O_3_ C_18_H_18_NO_7_S_3_ C_2_H_4_Cl_2_ H_2_SO_4_	0.0002 0.0005 0.0001 0.001 0.002

^a^Determined by Karl-Fischer method.

### Determination of binodal curves

For performing liquid-liquid extraction processes in a better manner binodal curve is required. In present work, the binodal curve for the studied systems has been determined by the cloud point method, as described in previous works^1,29^. In this method, the position of each point on the phase diagram is determined by adding titrant until the turbidity was observed. To determine the binodal curves, aqueous solutions of betaine at 50 wt% and aqueous solutions of phosphate salts or PEGDME_250_ at 50 wt% were used because at less than this concentration, the combination of these materials did not cause the formation of two phases. For this purpose, a sufficient amount of PEGDME_250_, K_3_PO_4_ or K_2_HPO4 solution of known concentrations was added drop by drop to the betaine solution until the two-phase region is observed. Then the distilled water was added to the mixture of components until clear and the homogenous solution is obtained. This procedure has been repeated to achieve enough data points for each system. Mass fraction of each component was determined using an analytical balance (Shimatzu, 321–34553, Shimatzu Co., Japan) with a precision of ± 1 × 10^−7^ kg.

### Determination of tie-lines

Sufficient accurate tie-line data are required to achieve optimal operating conditions for separation and extraction with high efficiency. In the present work to determine five tie-lines, a certain amount of betaine, PEGDME250 or phosphate salts (K_3_PO_4_ and K_2_HPO_4_) and deionized water in the biphasic region were mixed in 2-ml micro centrifuge tubes and vigorously stirred. For the system containing PEGDME250 2.27 g of polymer and about 0.94 g to 1.43 g of betaine was used together with a suitable amount of water. Similarly for the systems containing salts 1.37 g of each salt and about 0.95 g to 1.29 g of betaine together with appropriate amount of water was utilized. In the studied ATPSs the phase separation is quickly occurred within a few seconds. After centrifuging the prepared samples for 30 min they were left in the water bath to reach equilibrium at 298.15 K using thermostat (JULABO model MB, Germany) with a precision of 0.02 K. The time required for the two phases to separate completely from each other into two clear phases was about 48 h. When the desired two-phase systems were formed and completely separated, the concentration of betaine in the top and bottom phase was determined by UV spectroscopy. Since the betaine has less absorption in UV-visible spectrum and cannot be detected by a UV detector its identification and determination method is limited. In present work, we analyzed betaine by preparing its derivative using ethylene di-chloride as a solvent and blank and sulfuric acid in order to form corresponding sediment. For this purpose, first potassium triiodide solution was added to betaine solution; then the prepared mixture was covered and refrigerated overnight. This mixture was centrifuged at 3000 rpm for 10 min to sediment interfering periodides. Then the supernatant was added to centrifuged tubes. While it was placed inside the ice, a few drops of sulfuric acid was added to each tube and refrigerated for 2 h. After the mentioned period of time, the desired samples were centrifuged and the supernatant aspirated was removed with care so that the produced dark precipitate is not disturbed. Finally, the achieved precipitate was dissolved in ethylene dichloride and the absorption of the prepared solution was read in a spectrophotometer at 365 nm using ethylene di-chloride as a blank.

To quantify concentration of PEGDME_250_ in both phases, refractive index measurements performed at *T* = 298.15 K with the help of refractometer (ATAGO DR-A1, Japan) with a precision of ± 0.0001. The uncertainty in refractive index measurement is 0.0002. For dilute aqueous solutions consist of betaine and PEGDME_250_ or phosphate salts, the relation between the refractive index, *n*_D_ and the mass fraction of betaine, *w*_1_, and polymer or salt, *w*_2_is given by following Equation^30^:

1 $$n_D = n_w + a_1w_1+a_2w_2$$

Here, *n*_w_ is the refractive index of pure water for which we obtained the value of 1.3325 at *T* = 298.15 K. Two constants *a*_1_ and *a*_2_ relating to respectively betaine and polymer or salt and were achieved from the linear calibration plots of the refractive index of the corresponding binary solutions. The values of these constants and corresponding correlation coefficient values ${R^2}$ are given in Table 2. Since the Eq. 1 can only be used for dilute solutions, it was necessary for us to dilute the solutions to reach the desired concentrations. It was found that using Eq. (1) mass fraction of PEGDME250 can be determined with precision better than 0.002. The standard uncertainties in the a_1_ and a_2_values were achieved with the help of the method of least-squares to fit a linear curve to the refractive index calibration plots to estimate the parameters of the curve and their standard deviations. The concentration of phosphate salts in the top and bottom phases were determined by flame photometer (JENVEY model PFP7, England) with mass fraction uncertainty of 0.002^5^.

**Table 2:** Values of the parameters of Eq. 1, *a*_m_, for {betaine + PEGDME_250_ + H_2_O (w)} system.

Material	Constant	Value	C range (w/w)	^a^*R*^2^
PEGDME_250_	*a*_*P*_	0.1188	0 to 0.10	0.9997
Betaine	*a*_*b*_	0.1533	0 to 0.40	0.9997

^a^Where, *R*^2^, represented respective correlation coefficient value of the linear calibration plot of the refractive index against mass fraction of polymer and betaine at the mass fraction range (*C range*) of each material.

### Partition coefficient and the corresponding extraction efficiency

The primary tasks that must be done before partitioning studies are as follows. Five different ternary mixtures with the same compositions as used for tie-line measurements were prepared again in the biphasic region for each of the {[betaine + PEGDME_250_ + H_2_O]}, {[betaine + K_3_PO_4_ + H_2_O]} and {[betaine + K_2_HPO_4_ + H_2_O]} systems. After complete two phase formations of these mixtures as described in previous section, 2 mg of the desired drug has been added to each of the biphasic solutions at *T* = 298.15 K containing {betaine + PEGDME_250_ or salt + H_2_O} that have reached equilibrium. The prepared samples were then shaken vigorously for 30 min. Phase separation was observed after a few moments of stirring. In order to ensure the complete separation of the phases, centrifugation was performed for 20 min. At this stage, the prepared samples were placed in the mentioned water bath at 298.15 K and allowed to equilibrate overnight to achieve a complete drug partitioning between the two phases. After the samples reached equilibrium, a sample of each phase was withdrawn using a small syringe and analyzed for the drug content. The drug quantification, in both phases, were carried for acetaminophen, salicylic acid and ceftriaxone by UV spectroscopy at the wavelength of respectively, (243, 234 and 241) nm with the help of spectrophotometer (SPECORD 40- series Analytic Jena AG, Germany). To avoid interference from the phase components, the samples were diluted and analyzed against the blanks containing the same phase components but without drug.

Partition coefficient (*K*) and extraction efficiency (*E*) of drugs between the top and bottom phases were obtained by the following relations:

2 $$K=\frac{{w_{d}^{t}}}{{w_{d}^{b}}}$$

3 $$E=\frac{K}{{1+K}} \times 100$$

here $w_{d}^{t}$ and $w_{d}^{b}$ are equilibrium concentrations (in mass fraction) of the partitioned drugs, *d*, in the polymer or salt rich top, *t*, phase and the betaine rich bottom, *b*, phase, respectively.

## Determination of the tie-lines length and slope

The tie-line length, *TLL*, and slope, *S*, are two important values of the tie-lines that can be achieved, respectively, with the help of following Eqs:

4 $$TLL={\left[ {{{(w_{1}^{t} - w_{1}^{b})}^2}+{{(w_{2}^{t} - w_{2}^{b})}^2}} \right]^{0.5}}$$

5 $$S={{(w_{1}^{t} - w_{1}^{b})} \mathord{\left/ {\vphantom {{(w_{1}^{t} - w_{1}^{b})} {(w_{2}^{t} - w_{2}^{b})}}} \right. \kern-0pt} {(w_{2}^{t} - w_{2}^{b})}}$$

where, ${w_1}$ and ${w_2}$ indicating the equilibrium compositions (in mass fraction) of betaine (1) and salt or polymer (2), respectively.

## Results and discussion

**Fig. 1:**
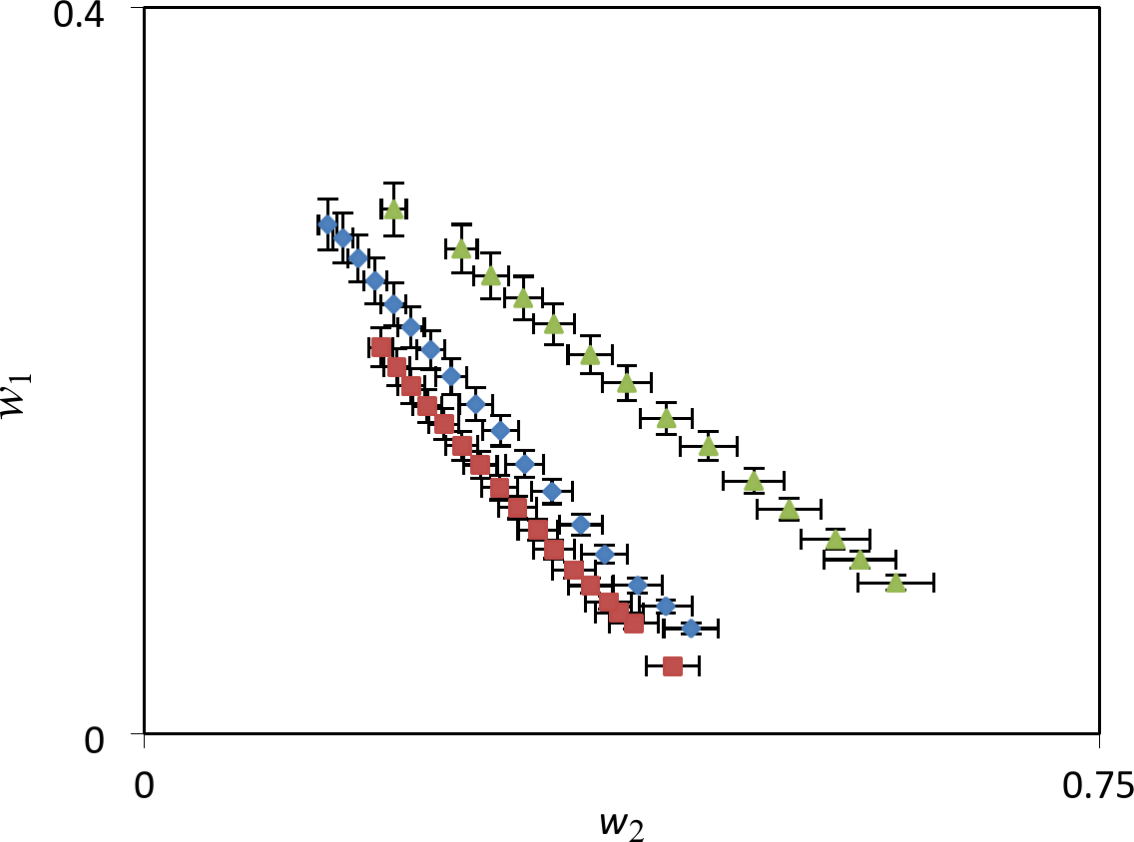
Comparison of binodal curves in mass fraction basis (*w*_i_) for different studied systems at *T* = 298.15 K: *Green traingle*, {betaine (1) + PEGDME_250_ (2) + H_2_O (3)}, *Red diamond*, {betaine (1) + K_3_PO_4_ (2) + H_2_O (3)}, *Blue square*, {betaine (1) + K_2_HPO_4_ (2) + H_2_O (3)}.

Prior to performing partitioning process, it is necessary to obtain the phase diagram that separates the single-phase region from the two-phase one, which is called a binodal curve in the scientific nomenclature. The obtained binodal data for the ternary systems {betaine + PEGDME_250_ + H_2_O}, {betaine + K_3_PO_4_ + H_2_O} and{betaine + K_2_HPO_4_+ H_2_O} at *T*= (298.15, 308.15 and 318.15) K and ambient pressure (≈ 85 kPa) are given in Tables 3, 4, and 5. Since the salting-out ability of a polymer or a salt is important and effective in carrying out the separation process, in Fig. 1, we have shown the ability of all three systems in terms of the formation and extent of the two-phase region at *T*= 298.15 K by plotting the binodal curves. According to the position of these curves, it is clear that in regard with the two-phase formation salts are more suitable than the polymer in the studied systems and therefore the ATPSs containing phosphate salts and betaine may act as a good candidate for drug partitioning. From previous studies^23^, it was found that the value of Gibbs energy of hydration (∆G_hyd_) of the salt plays a significant role in the stability of the two-phase system so that as this quantity becomes more negative the formed two-phase system will be more stable. In the studied systems, the PO4^-3^anion (∆G_hyd_=- 2835 kJ. mol^-1^) has a more negative value compared to the HPO4^-2^ (∆G_hyd_=-1125 kJ. mol^-1^)anion. Therefore, the PO4^-3^anion will form a more stable two-phase system and expected to have higher extraction efficiency. These values of ∆G_hyd_have been obtained in previous works^23^through the Marcus method^31^

**Table 3:** Experimental binodal data in mass fraction, *w*_i_, for {betaine (1) + PEGDME_250_ (2) + H_2_O (3)} ATPS at *T* = (298.15, 308.15, 318.15) K and *P* ≈ 85 kPa.^a^.

T / K = 298.15		T / K = 308.15		T / K = 318.15	
100. ^*b*^*w*_1_	100. ^*b*^*w*_2_	100. *w*_1_	100. *w*_2_	100. *w*_1_	100. *w*_2_
Betaine/PEGDME_250_					
8.33	59.00	6.76	60.45	5.93	61.29
9.60	56.19	8.12	57.48	7.42	58.03
10.72	54.26	9.41	55.31	8.76	55.10
12.37	50.62	11.27	51.41	10.67	50.49
13.93	47.84	12.88	48.03	12.43	46.95
15.85	44.29	14.82	44.04	14.45	42.42
17.38	40.99	16.57	40.95	16.2	38.99
19.33	37.88	18.40	37.24	18.14	35.33
20.87	35.00	20.08	34.31	19.76	32.30
22.57	32.15	21.76	31.29	21.24	29.08
23.99	29.74	23.16	28.81	22.95	25.95
25.23	27.21	24.29	26.51	24.08	24.16
26.71 28.88	24.88 19.58	26.01 27.28	23.41 19.98	26.26 27.54	20.14 18.12

^**a**^ The standard uncertainties ***u*** for mass fraction, temperature and pressure are: ***u*** (***w***_**i**_) = 0.002; ***u*** (***T***) = 0.05 K and ***u***(*P*) = 0.5 kPa, respectively.

^**b**^*w*_1_ and *w*_2_ represent mass fractions of betaine and the poly ethylene glycol dimethyl ether, respectively.

**Table 4:** Experimental binodal data in mass fraction, *w*_i_, for {betaine (1) + K_2_HPO_4_ (2) + H_2_O (3)} ATPS at *T* = (298.15, 308.15, 318.15) K and *P* ≈ 85 kPa.^a^.

T / K = 298.15		T / K = 308.15		T / K = 318.15	
100. ^*b*^*w*_1_	100. ^*b*^*w*_2_	100. *w*_1_	100. *w*_2_	100. *w*_1_	100. *w*_2_
Betaine/K_2_HPO_4_					
5.81	42.92	4.78	42.96	4.72	40.51
7.03	40.93	5.40	41.45	7.38	36.26
8.18	38.73	8.23	37.50	9.98	32.08
9.88	36.14	8.99	36.00	12.16	29.84
11.52	34.27	10.52	33.82	13.18	28.45
13.34	32.00	12.59	30.81	14.54	26.68
14.85	29.87	14.95	27.55	15.48	24.91
16.69	27.95	17.56	24.14	17.11	23.12
18.15	26.01	20.09	20.90	18.53	21.54
19.69	24.08	22.48	18.14	19.64	19.97
21.14	22.48			21.35	18.09
22.39	20.92			22.63	16.25
23.65 24.94 26.18 27.29 28.04	19.56 18.11 16.79 15.59 14.38			23.45 24.41 25.16	15.46 14.31 13.63

^**a**^The standard uncertainties ***u*** for mass fraction, temperature and pressure are: ***u*** (***w***_**i**_) = 0.002; ***u*** (***T***) = 0.05 K and ***u***(*P*) = 0.5 kPa, respectively.

^**b**^*w*_1_ and *w*_2_ represent mass fractions of betaine and K_2_HPO_4_ salt, respectively.

**Table 5:** Experimental binodal data in mass fraction, *w*_i_, for {betaine (1) + K_3_PO_4_ (2) + H_2_O (3)} ATPS at *T* = (298.15, 308.15, 318.15) K and *P* ≈ 85 kPa.^a^.

T / K = 298.15		T / K = 308.15		T / K = 318.15	
100. ^*b*^*w*_1_	100. ^*b*^*w*_2_	100. *w*_1_	100. *w*_2_	100. *w*_1_	100. *w*_2_
Betaine/ K_3_PO_4_					
3.73 6.09 6.68 7.26 8.17 9.02 10.16 11.23 12.47 13.57 14.81 15.87 17.06 18.05 19.17 20.20 21.28 22.13	41.48 38.41 37.28 36.44 35.02 33.71 32.14 30.88 29.3 27.89 26.37 24.92 23.52 22.2 20.93 19.81 18.57 17.43	5.42 6.34 7.75 8.38 9.46 10.67 11.86 13.26 14.35 15.42 16.43 18.25 19.72 22.71	37.56 36.45 34.68 33.49 31.37 30.16 28.43 27.52 25.96 24.79 22.84 20.68 19.56 15.62	5.71 7.22 8.50 9.27 11.54 12.66 13.83 14.92 16.19 17.18 18.25 19.28 20.34 22.07 23.16 23.92 25.61	36.34 34.54 32.72 31.11 28.33 26.96 25.49 23.97 22.58 21.24 19.95 18.87 17.66 15.41 14.47 13.89 12.62

^**a**^The standard uncertainties ***u*** for mass fraction, temperature and pressure are: ***u*** (***w***_**i**_) = 0.002; ***u*** (***T***) = 0.05 K and ***u***(*P*) = 0.5 kPa, respectively.

^**b**^*w*_1_ and *w*_2_ represent mass fractions of betaine and K_3_PO_4_ salt, respectively.

## Effect of temperature on the phase separation

To examine the temperature effect on the phase diagrams of the studied systems, experimental binodal curves for ternary betaine-based aqueous systems containing either a polymer, potassium phosphate (K_3_PO_4_), or potassium hydrogen phosphate (K₂HPO₄) were plotted, as shown in Figures S1−3in the supporting information. These binodal curves represent the boundary between single-phase and two-phase regions in the phase diagram. The results indicate that for all three systems those containing polymer, K_3_PO_4_, or K_2_HPO_4_an increase in temperature causes an expansion in the area of the two-phase region. This expansion means that the system more readily separates into two distinct phases as the temperature increases, although this effect is not very large. In the case of the aqueous two-phase system (ATPS) containing the polymer, the temperature increase leads to the breakdown of hydrogen bonds between PEGDME250 (a polyethylene glycol dimethyl ether) and water molecules. PEGDME250 forms hydrogen bonds with water, stabilizing the system in a single phase. When these hydrogen bonds break at higher temperatures, the hydrophobicity of PEGDME250 increases. This makes the polymer less soluble in water, which facilitates easier phase separation. The hydrophobic interactions dominate at higher temperatures, causing the polymer-rich phase to separate more readily from the aqueous phase. In systems containing salts like K₃PO₄ or K₂HPO₄, the temperature can influence the solubility of salts and the interactions between water molecules and ions, thus affecting the overall phase behavior. Salt-based aqueous two-phase systems (ATPS) are driven by the salting-out effect, where salts induce phase separation by reducing the solubility of certain solutes in water. The expansion of the two-phase region with temperature suggests that higher temperatures enhance the salting-out effect, leading to easier phase separation^32–34^.

## Binodal data and correlation

The obtained binodal data presented in Tables 3, 4 and 5were fitted with the help of non-linear least-squares regression method to the Merchuk, Eq. (6)^15^, Zafarani-Moattar and Nemati, Eq. (7)^24^and the modified effective excluded volume theory Eq. (8)^25^. These relations are as follows:

6 $$w_1=a \:exp (bw_2^{0.5}- cw_2^3)$$

7 $$w_1=\alpha+\beta ln (w_2) + \gamma w_2$$

8 $$\text{l}\text{n}\left(A\frac{{w}_{1}}{{M}_{1}}+B\right)+A\frac{{w}_{2}}{{M}_{2}}=0$$

In Eqs. 6 to 8, *w*_1_ and *w*_2_ are the mass fractions of betaine and salts or polymer, respectively. The fitting parameters of Eq. (6), (*a*, *b* and *c*), Eq. (7), (*α*, *β*, and *γ*) and Eq. (8), (*A* and *B*) are obtained from the correlation of the experimental binodal data presented in Tables 3, 4 and 5 at different temperatures and the results together with the standard deviations are respectively given in Tables 6, 7 and 8. The obtained standard deviation, *sd*, values ​​indicate that the mentioned relationships can be used effectively to reproduce binodal data. However, using Eq. (8) very small *sd* values (as reported in Table 8) show that the modified effective excluded volume theory with only two parameters produce a better result than the other two frequently used equations havening three parameters.

**Table 6:** Values of parameters of equation, Eq. (6), for {betaine + PEGDME_250_ or salt + H_2_O) ATPSs at different temperatures.

T / K	a	b	c	_100 sd_^a^	
Betaine + K_2_HPO_4_ + H_2_O					
298.15	0.695	−1.989	22.896	0.14	
308.15	0.660	−1.952	33.528	0.21	
318.15	0.626	−1.980	34.241	0.29	
Betaine + K_3_PO_4_ + H_2_O					
298.15	0.599	−1.827	35.449	0.23	
308.15	0.599	−1.958	34.885	0.32	
318.15	0.618	−2.188	31.802	0.26	
Betaine + PEGDME_250_ + H_2_O					
298.15	1.002	−1.791	25.669	0.39	
308.15	0.969	−1.770	30.267	0.33	
318.15	1.023	−2.047	31.906	0.23	

^*a*^$sd=(\sum\nolimits_{{i=1}}^{N} {{{(w_{i}^{{cal}} - w_{i}^{{\exp }})}^2}/N{)^{0.5}}}$, where *w*_i_ and *N* represented mass fraction of polymer or salt and number of binodal data, respectively.

**Table 7:** Values of parameters of equation, Eq. (7), for {betaine + PEGDME_250_ or salt + H_2_O) ATPSs at different temperatures.

T / K	α	β	γ	_100 sd_^a^
Betaine + K_2_HPO_4_ + H_2_O				
298.15	0.350	−0.046	−0.937	0.16
308.15	0.374	−0.035	−1.098	0.17
318.15	0.354	−0.032	−1.054	0.20
Betaine + K_3_PO_4_ + H_2_O				
298.15	0.378	−0.024	−1.090	0.15
308.15	0.352	−0.027	−1.039	0.32
318.15	0.278	−0.048	−0.875	0.26
Betaine + PEGDME_250_ + H_2_O				
298.15	0.669	−0.024	−1.717	0.33
308.15	0.674	−0.019	−1.807	0.28
318.15	0.535	−0.061	−1.589	0.21

^*a*^$sd=(\sum\nolimits_{{i=1}}^{N} {{{(w_{i}^{{cal}} - w_{i}^{{\exp }})}^2}/N{)^{0.5}}}$, where *w*_i_ and *N* represented mass fraction of polymer or salt and number of binodal data, respectively.

**Table 8:** Values of parameters of equation, Eq. (8), for {betaine + PEGDME_250_ or salt + H_2_O) ATPSs at different temperatures.

T/K	A	B	*R*^2^	_100 sd_^a^
Betaine + PEGDME250 + H_2_O				
298.15	−0.8353	1.2741	0.9997	0.02
308.15	−0.6020	1.1899	0.9998	0.03
318.15	−0.3511	1.1062	0.9999	0.06
Betaine + K_2_HPO4 + H_2_O				
298.15	−2.8545	1.8787	0.9975	0.06
308.15	−1.8008	1.5006	0.9991	0.02
318.15	−2.7712	1.7708	0.9976	0.05
Betaine + K_3_PO_4_ + H_2_O				
298.15	−0.8062	1.2332	0.9998	0.01
308.15	−1.0223	1.2902	0.9996	0.01
318.15	−1.3934	1.3957	0.9993	0.03

^*a*^$sd=(\sum\nolimits_{{i=1}}^{N} {{{(w_{i}^{{cal}} - w_{i}^{{\exp }})}^2}/N{)^{0.5}}}$, where *w*_i_ and *N* represented mass fraction of polymer or salt and number of binodal data, respectively.

## Estimated plait point (critical concentration)

Plait point is an important characteristic of the phase diagram where the two liquid phases become identical. The location of the plait point for studied systems is obtained by the help of the following Eq. 

9 $${w_1}=f+g{w_2}$$

**Fig. 2:**
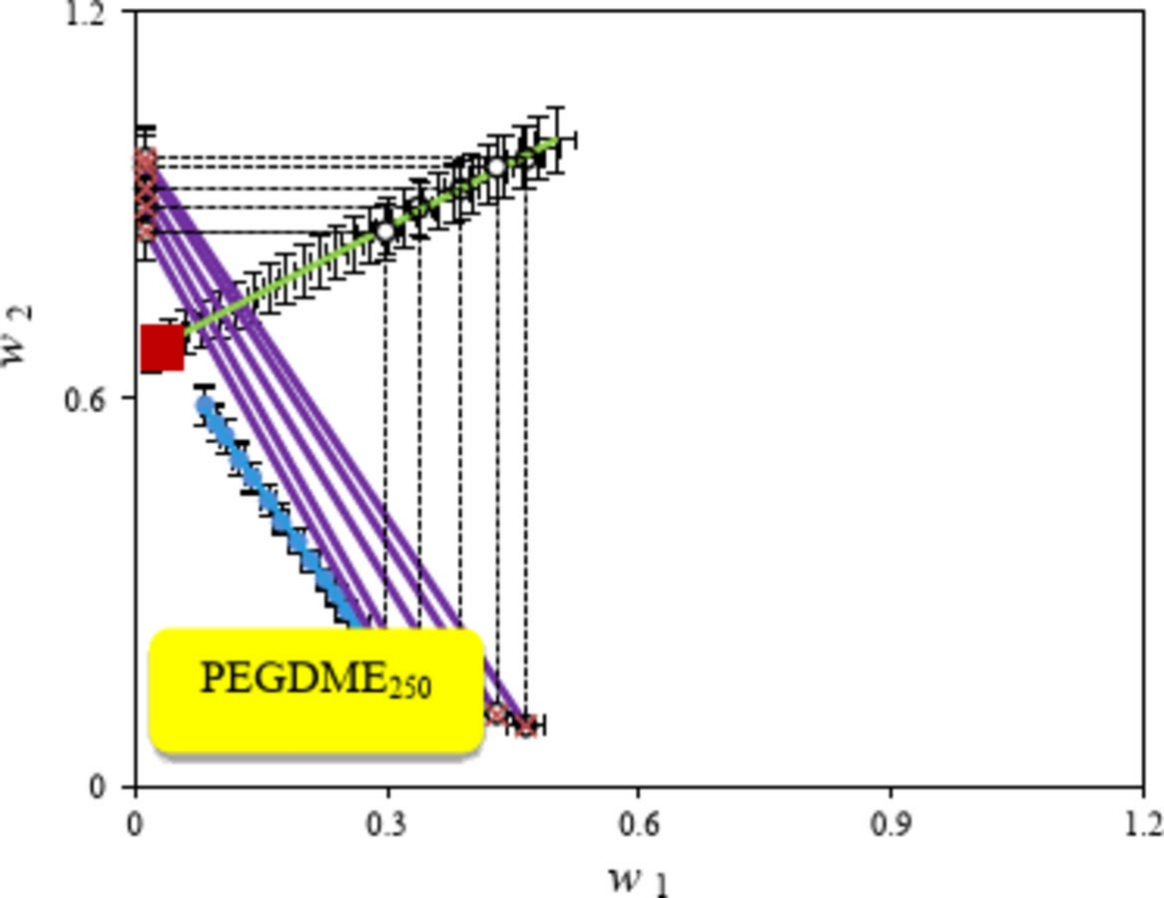
Binodal curve, tie-lines and plait point for the {betaine (1) + PEGDME_250_ (2) + H_2_O (3)} system at *T* = 298.15 K: (*Blue circle*) experimental binodal data, (*Blue* *continuous line*) calculated binodal from Eq. (7), (*open circle with dotted line*) calculated auxiliary curves, (*continuous dark blue line*) tie-lines data, (* continuous dark blue line with cross*) calculated from Eq. (12) and *Red square* plait point.

Here, *f* and *g* indicate the fitting parameters. The extrapolation from the auxiliary curves were fitted to Eq. (9) to estimate plait point. The results are reported in Table 9. As an example, the locus of the estimated plait point for the {betaine + PEGDME_250_ + H_2_O} system is illustrated in Fig. 2. Similar plots for the aqueous betaine ATPSs containing K_3_PO_4_ and K_2_HPO_4_ are shown in Figures S 4 and S 5 in supporting information.

**Table 9:** Values of parameters of Eq. (9), (*f*,* g*) and the plait points, for the {betaine + PEGDME_250_ or salt + H_2_O) system at *T* = 298.15 K.

System	*f*	*g*	Plait Point (w_1_, w_2_, w_w_) a
Betaine + PEGDME_250_ + water	66.464	0.673	(0.0322, 0.6798, 0.2880)
Betaine + K_3_PO_4_ + water	34.922	0.564	(0.0759, 0.3899, 0.5342)
Betaine + K_2_HPO_4_ + water	33.919	0.654	(0.0657, 0.3798, 0.5544)

^*a*^*w*_1_, *w*_2_ and *w*_*w*_ are mass fractions of betaine, PEGDME_250_ or salt and water.

## Tie-line data and correlation

**Fig. 3:**
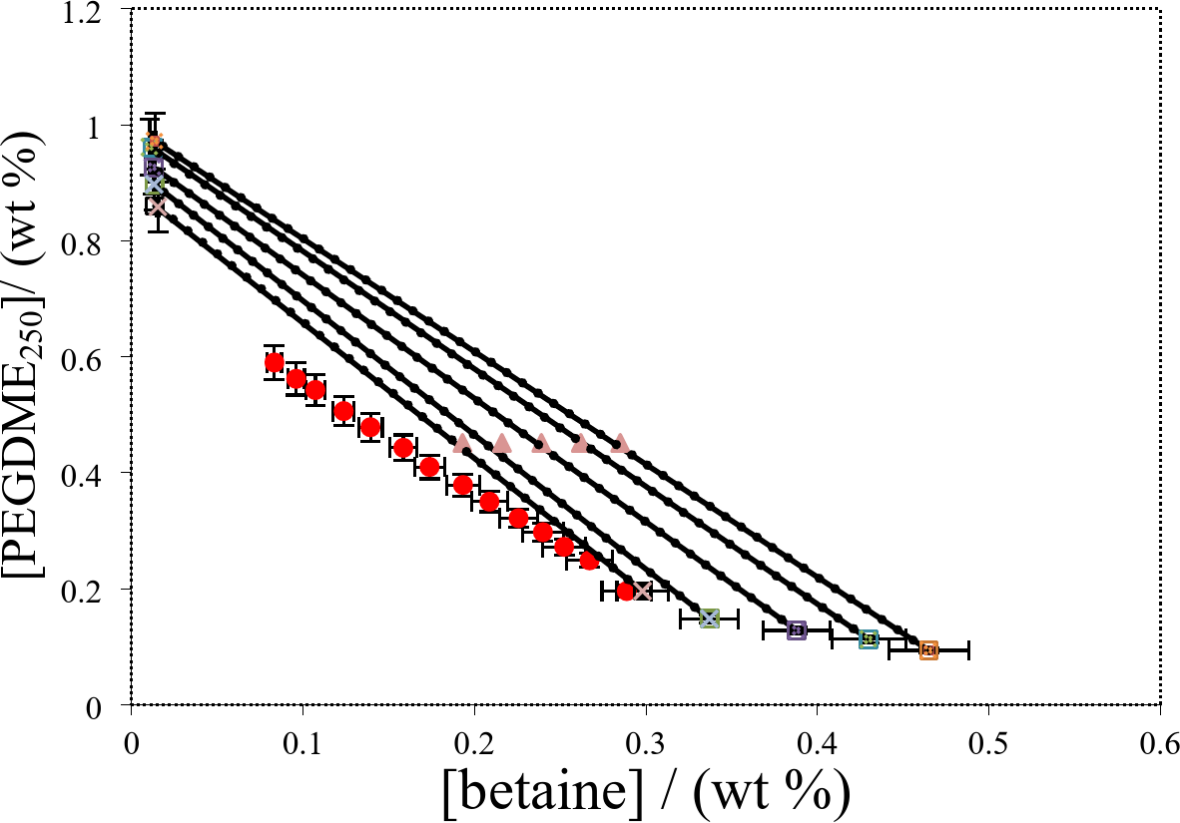
Phase diagram for {betaine (1) + PEGDME_250_ (2) + H_2_O (3)} system at *T* = 298.15 K: * Red circle*, binodal data; * Blue triangle*, the initial composition of the tie line; (*Blue circle with line*) experimental tie-lines; (*Green triangle with dotted line*) calculated tie-lines using Steschenow type equation Eq. (12).

**Fig. 4:**
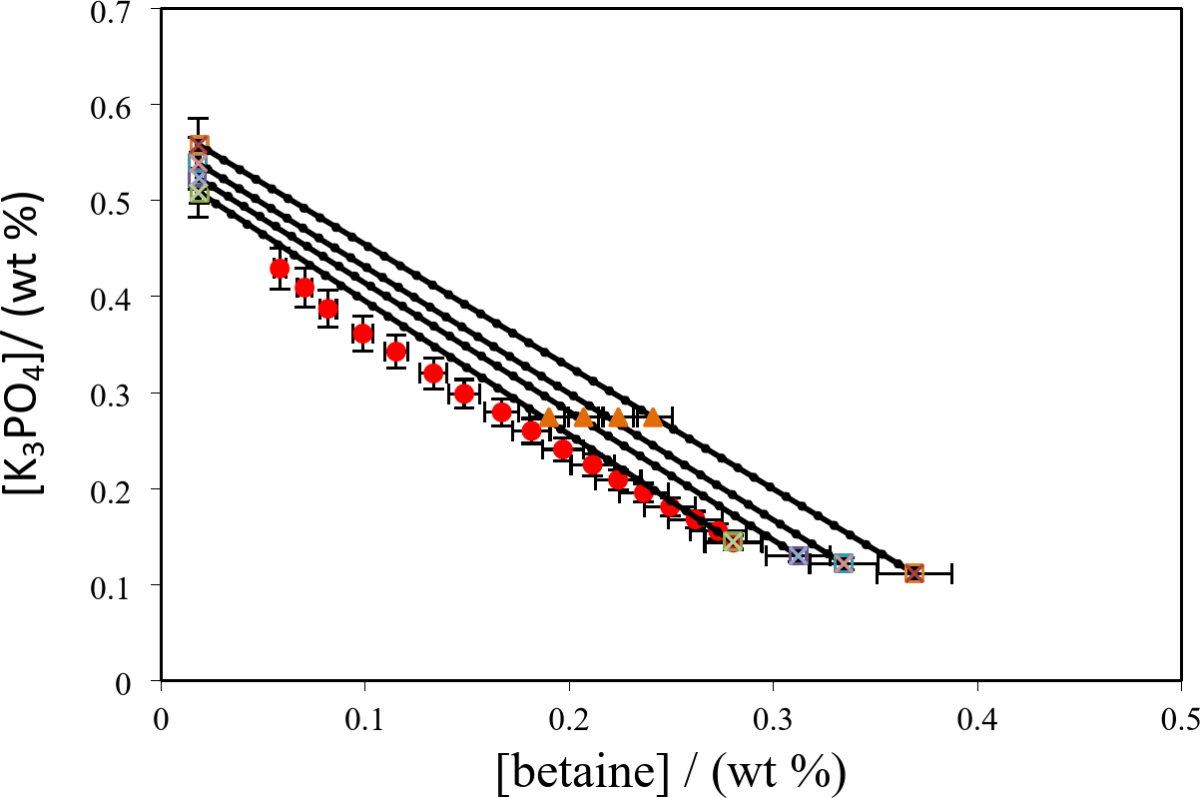
Phase diagram for {betaine (1) + K_3_PO_4_ (2) + H_2_O (3)} system at *T* = 298.15 K: * Red circle*, binodal data; * Blue triangle*, the initial composition of the tie line; (*Blue circle with line*) experimental tie-lines; (*Green triangle with dotted line*) calculated tie-lines using Steschenow type equation Eq. (13).

**Fig. 5:**
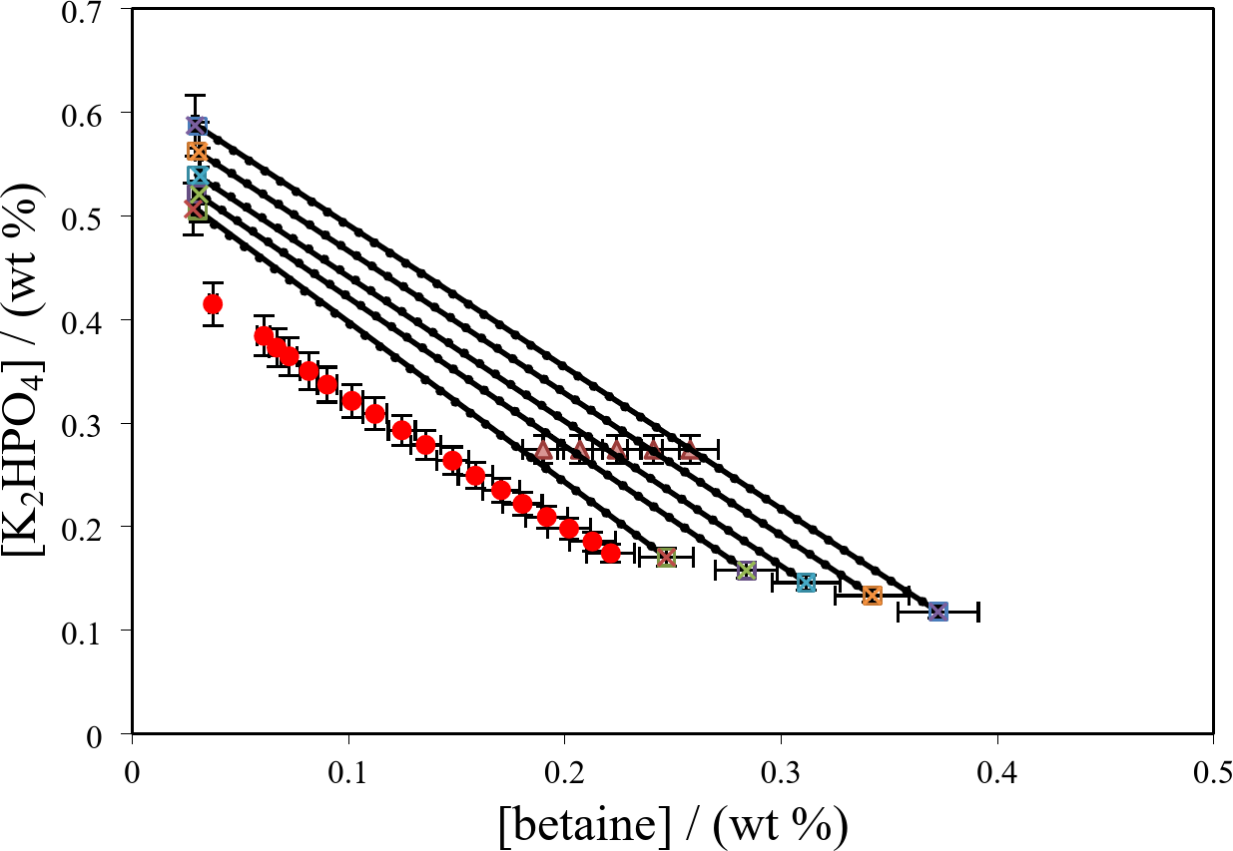
Phase diagram for {betaine (1) + K_2_HPO_4_ (2) + H_2_O (3)} system at *T* = 298.15 K: * Red circle*, binodal data; *Blue triangle*, the initial composition of the tie-lines; (*Blue circle with line*) experimental tie-lines; (*Green traingle with dotted line*) calculated tie-lines using Steschenow type equation (Eq. (12).

The experimental tie-lines for the betaine based ATPSs composed of PPGDME_250_, K_3_PO_4_ and K_2_HPO_4_) are presented in Figs. 3, 4 and 5. The corresponding tie-line data are presented in Table 10. To regenerate tie-lines for the investigated systems the experimental tie-lines of Table 10 were fitted to the Othmer-Tobias, Bancraft^26,27^and Setschenow–type^28^ equations at 298.15 K.

**Table 10:** Experimental tie-line data together with the corresponding tie-line length (*TLL*) and slope for {betaine + PEGDME_250_ or salts (K_3_PO_4_, K_2_HPO_4_) + H_2_O} systems at 298.15 K and atmospheric pressure (≈ 85 kPa).^a^

Overall composition / wt %		Betaine-rich phase composition / wt %		Polymer-rich phase composition / wt %			
Betaine	PEGDME_250_	Betaine	PEGDME_250_	Betaine	PEGDME_250_	*TLL*	Slope
19.3	45.129	29.8	16.94	1.33	86.0	74.69	−0.412
21.6	45.129	33.7	15.97	1.32	89.7	80.52	−0.439
23.9	45.129	38.8	13.80	1.30	92.6	87.26	−0.476
26.2	45.129	43.0	10.98	1.26	96.0	94.71	−0.491
28.5	45.129	46.5	9.29	1.24	97.2	98.87	−0.515
Betaine-rich phase composition / wt%				Salt-rich phase composition / wt %			
Betaine	K_2_PO_4_	K_2_PO_4_	Betaine	K_2_PO_4_	Betaine		
19.0	27.436	14.47	28.02	50.81	1.90	44.70	−0.719
20.7	27.436	13.00	31.22	52.41	1.77	49.19	−0.747
22.4	27.436	12.18	33.45	53.91	1.79	52.38	−0.759
24.1	27.436	11.16	36.91	55.77	1.87	56.72	−0.785
Betaine	K_3_PO_4_	K_3_PO_4_	Betaine	K_3_PO_4_	Betaine		
19.0	27.436	17.04	24.70	50.54	3.03	39.89	−0.647
20.7	27.436	15.78	28.40	52.11	3.00	44.32	−0.699
22.4	27.436	14.60	31.16	53.94	2.97	48.39	−0.717
24.1	27.436	13.35	34.22	56.24	2.99	53.05	−0.728
25.8	27.436	11.78	37.26	58.64	3.04	58.02	−0.730

^*a*^Standard uncertainties for mass fraction, temperature and pressure are *u* (*w*i) = 0.002, *u* (*T*) = 0.05 K and *u* (*p*) = 0.5 kPa, respectively. Tie-line length (*TLL*) and slope at different concentrations were calculated from Eqs. (5) and (6).

### Othmer-Tobias and Bancraft equations

In reproducing and examining reliability of tie-lines Othmer-Tobias, Eq. (10), and Bancraft, (Eq. 11), have been frequently used.

10 $$\frac{1-{w}_{2}^{t}}{{w}_{2}^{t}}=k(\frac{1-{w}_{2}^{b}}{{w}_{2}^{b}})n$$

11 $$(\frac{{w}_{w}^{b}}{{w}_{2}^{b}})=k_1(\frac{{w}_{w}^{t}}{{w}_{1}^{t}})r$$

The fitting parameters (*k*, *n*, *k*_1_ and *r*) along with the associated standard deviations (*R*^2^) are listed in Table 11. It was found that there is a linear relationship between the logarithm of two sides in Eqs. (10, 11), (i.e., $\:\text{l}\text{o}\text{g}\left[\right(1-{w}_{1}^{t})/{w}_{1}^{t}$ plots against $\:\:\text{l}\text{o}\text{g}\left[\right(1-{w}_{2}^{b})/{w}_{2}^{b}]\:$ and $\:\text{l}\text{o}\text{g}\left[\right({w}_{w}^{b}/{w}_{s}^{b}\left)\right]$ against$\:\:\:\text{l}\text{o}\text{g}\left[\right({w}_{w}^{t}/{w}_{1}^{t}\left)\right]$) which indicates the acceptability of the obtained tie-lines. Also, from the reported rather small deviations (*Dev*) in Table 11 we conclude that Eqs. (10) and (11) can be satisfactorily applied to correlate the tie-lines of mentioned systems.

**Table 11:** Values of parameters of Othmer-Tobias, Bancroft, (*k*, *n*, *k*_1_, *r*) and for {betaine + PEGDME_250_ or salts (K_3_PO_4_, K_2_HPO_4_) + H_2_O} at *T* = 298.15 K.

Othmer-Tabias and Bancroft equations (Eqs. (10) and (11))					
	*K*	*n*	*k*_1_	*r*	^*a*^ *Dev*
Betaine + PEGDME_250_ +water Betaine + K_3_PO_4_ + water Betaine + K_2_HPO_4_ + water	63.089 2.723 3.062	0.102 2.032 1.850	4.987 0.632 0.559	−0.227 0.542 0.603	0.09 0.06 0.07

^*a*^$Dev=\sum\nolimits_{{p}}\sum\nolimits_{{l}}\sum\nolimits_{{j}}\sum\nolimits_{{T}} {{{((100w_{p,l,j,T}^{{cal}} - 100w_{p,l,j,T}^{{exp }})}^2}/6N{)}}$ , where *w*_*p, l,j, T*_ is the mass fraction of the component *j* (i.e. betaine, polymer, salt or water) in the phase *p* for *l*th tie-line at temperature *T* and *N* represents the number of tie-line data points. . .

#### Setschenow-type equation

In correlation of tie-lines data the following simple Setschenow Equation^28^ with only two parameters was also used from which the salting-out ability in the studied ATPS systems may be examined:

12 $$\text{ln}(\frac{m_1^t}{m_1^b})=k_c+k_s({m_2^b}-{m_2^t})$$

where, is the salting-out coefficient, *k*_c_ is a constant and *m*_1_ and *m*_2_ are the molality of betaine and polymer or salt, respectively. The experimental liquid-liquid equilibrium data of Table 10 also were fitted to Eq (13) using the following objective function:

13 $$Of=\sum\limits_{T} {\sum\limits_{P} {\sum\limits_{l} {\sum\limits_{j} {\left( {w_{{T,P,l,j}}^{{cal}} - w_{{T,P,l,j}}^{{\exp }}} \right)} } } }$$

where, ${w_{T,P,l,j}}$ represents the mass percent of the component *j* in the phase (betaine, polymer or salt, and water) for *l*th tie-line at working temperature. The superscripts “*cal*” and “*exp*” indicated the calculated and experimental values, respectively. The corresponding correlation coefficient values, *R*^2^, together with the fitted parameters (*k*_s_, *k*_c_) and deviations are listed in Table 12. Furthermore, the parameters given in Table 12 has been used to examine the performance of Eq. (12) in representing the tie-line data of the investigated systems. The obtained deviations presented in Table 12 show that Eq. (12) can be accurately used to reproduce the tie-line data of the investigated systems. The good capability of Eq. (12) can be seen in a better manner in Figs. 3, 4 and 5 where the comparison between the experimental and correlated tie-lines is also shown for the aqueous {betaine + PEGDME_250_ or salts + H_2_O} at 298.15 K.

**Table 12:** Values of parameters Setschenow type equation, (*k*_c_, *k*_s_) (kg·K·mol^−1^), for {betaine + PEGDME_250_ or salts (K_3_PO_4_, K_2_HPO_4_) + H_2_O} at 298.15 K.

Setschenow type equation (Eq. 12)			
System	*k*_c_	*k*_s_	*Dev* ^*a*^
PEGDME_250_	−1.7450	−0.0062	0.08
K_2_HPO_4_	1.4341	0.1117	0.04
K_3_PO_4_	1.7431	0.2051	0.03

^*a*^$Dev=\sum\nolimits_{{p}}\sum\nolimits_{{l}}\sum\nolimits_{{j}}\sum\nolimits_{{T}} {{{((100w_{p,l,j,T}^{{cal}} - 100w_{p,l,j,T}^{{exp }})}^2}/6N{)}}$ , where *w*_*p, l, j, T*_ is the mass fraction of the component *j* (i.e. betaine, polymer, salt or water) in the phase *p* for *l*th tie-line at temperature *T* and *N* represents the number of tie-line data points.

The obtained slopes of the lines (*k*s values) are in agreement with experimental observations in which ATPS formation with phosphate salts occurs at lower levels of the betaine and salt than ATPS containing betaine and the polymer. When we compare salting-out ability of two phosphate salts, since the ATPS containing K_3_PO_4_ has a bigger *k*s value than the K_2_HPO_4_ easier ATPS formation is occurred with K_3_PO_4_.

From the standard deviations tablets in Table 12 it can be said with confidence that the both models applied at *T* = 298.15 K have good performances for corresponding LLE data in three systems and among the all models used; Setschenow model is the best in representing the tie-line data of investigated systems.

### The performance ATPSs composed of betaine and PPGDME or phosphate salts in the partition behavior of drugs

**Fig. 6:**
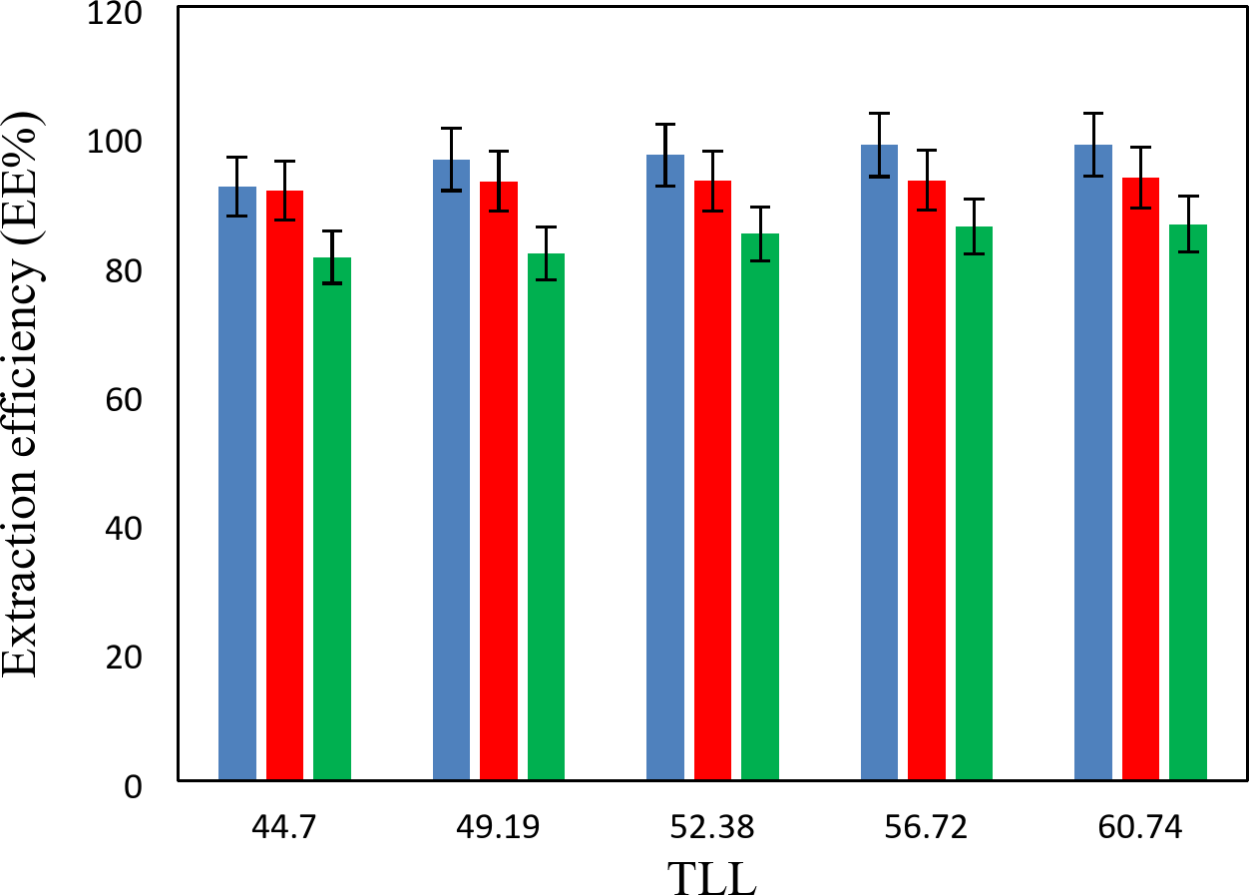
Effect of system type on extraction efficiency (*EE* %) for salicylic acid at *T* = 298.15 K: *Orange square*, {betaine + PEGDME_250_ + H_2_O}; * Green square*, {betaine + K_2_HPO_4_ + H_2_O}; * Blue square*, {betaine + K_3_PO_4_ + H_2_O}.

**Fig. 7:**
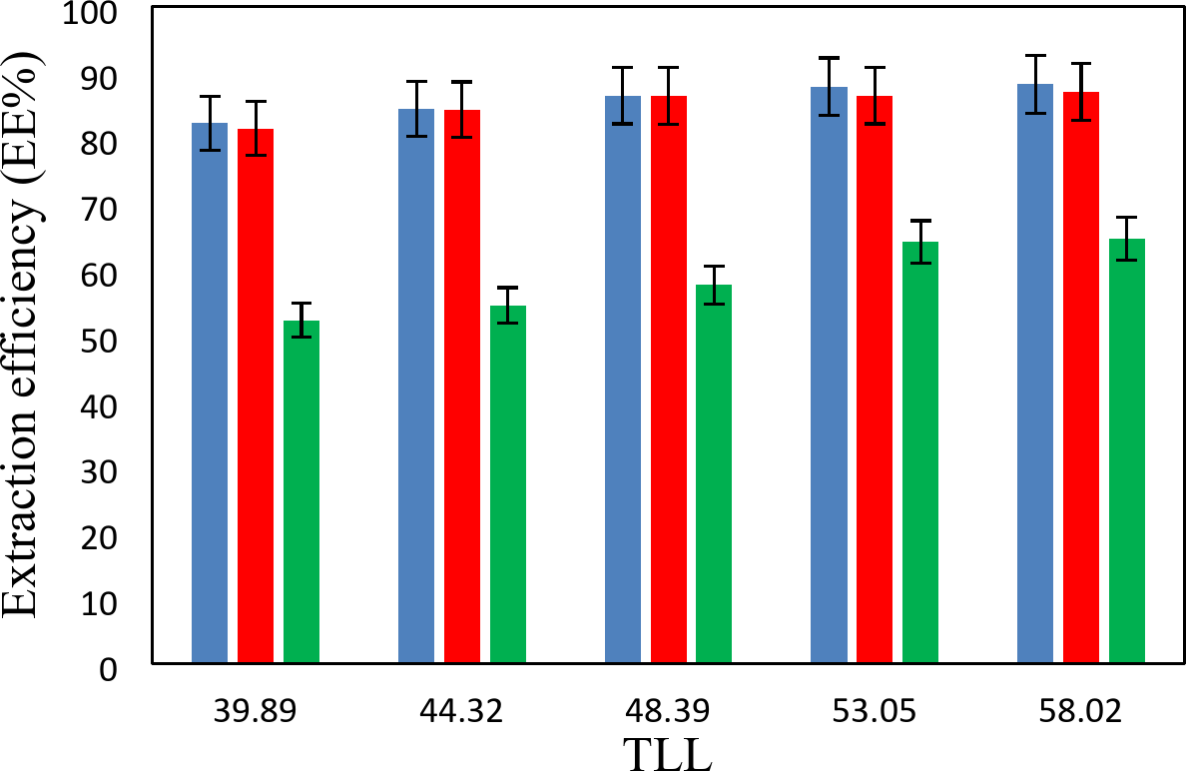
Effect of system type on extraction efficiency (*EE*%) for acetaminophen at *T* = 298.15 K: * Orange square*, {betaine + PEGDME_250_ + H_2_O}; * Green square*, {betaine + K_2_HPO_4_ + H_2_O}; * Blue square*, {betaine + K_3_PO_4_ + H_2_O}.

**Fig. 8:**
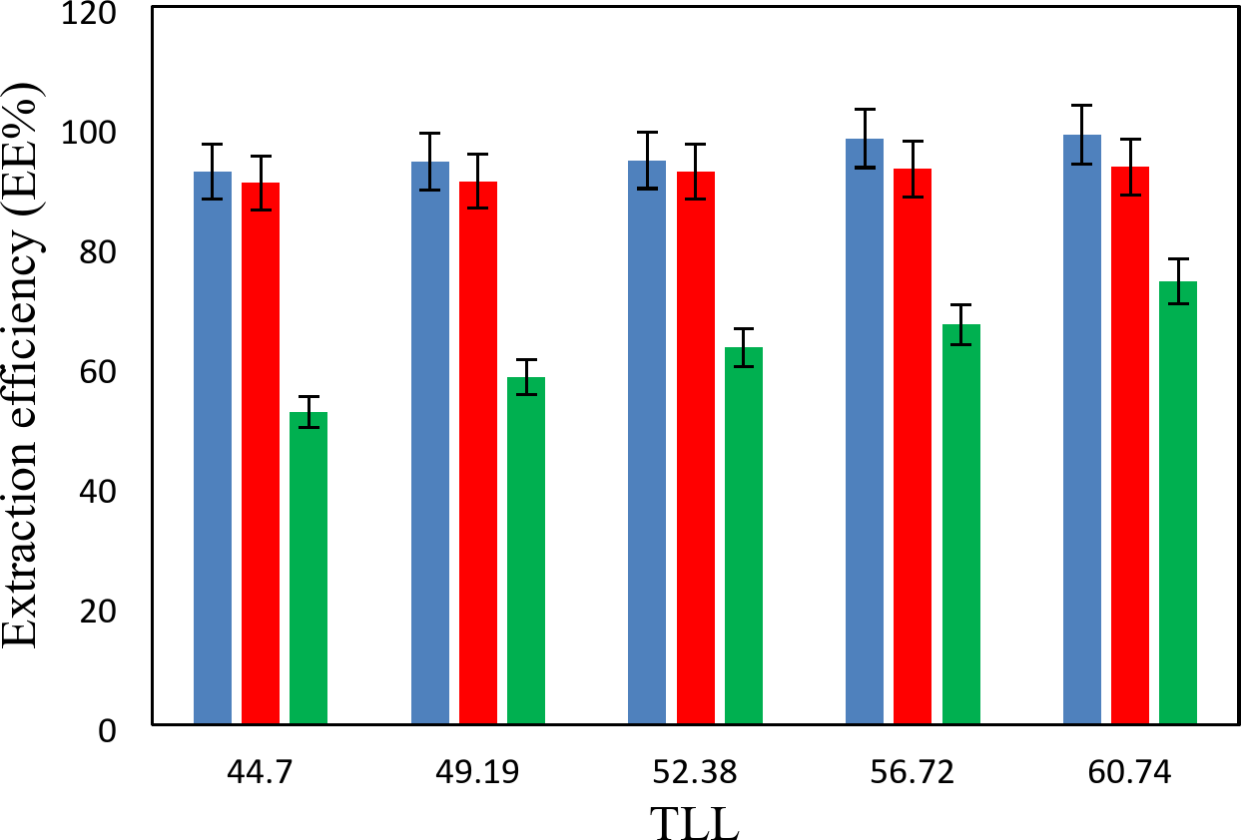
Effect of system type on extraction efficiency (*EE* %) for ceftriaxone at *T* = 298.15 K: * Orange square*, {betaine + PEGDME_250_ + H_2_O}; * Green square*, {betaine + K_2_HPO_4_ + H_2_O}; * Blue square*, {betaine + K_3_PO_4_ + H_2_O}.

**Fig. 9:**
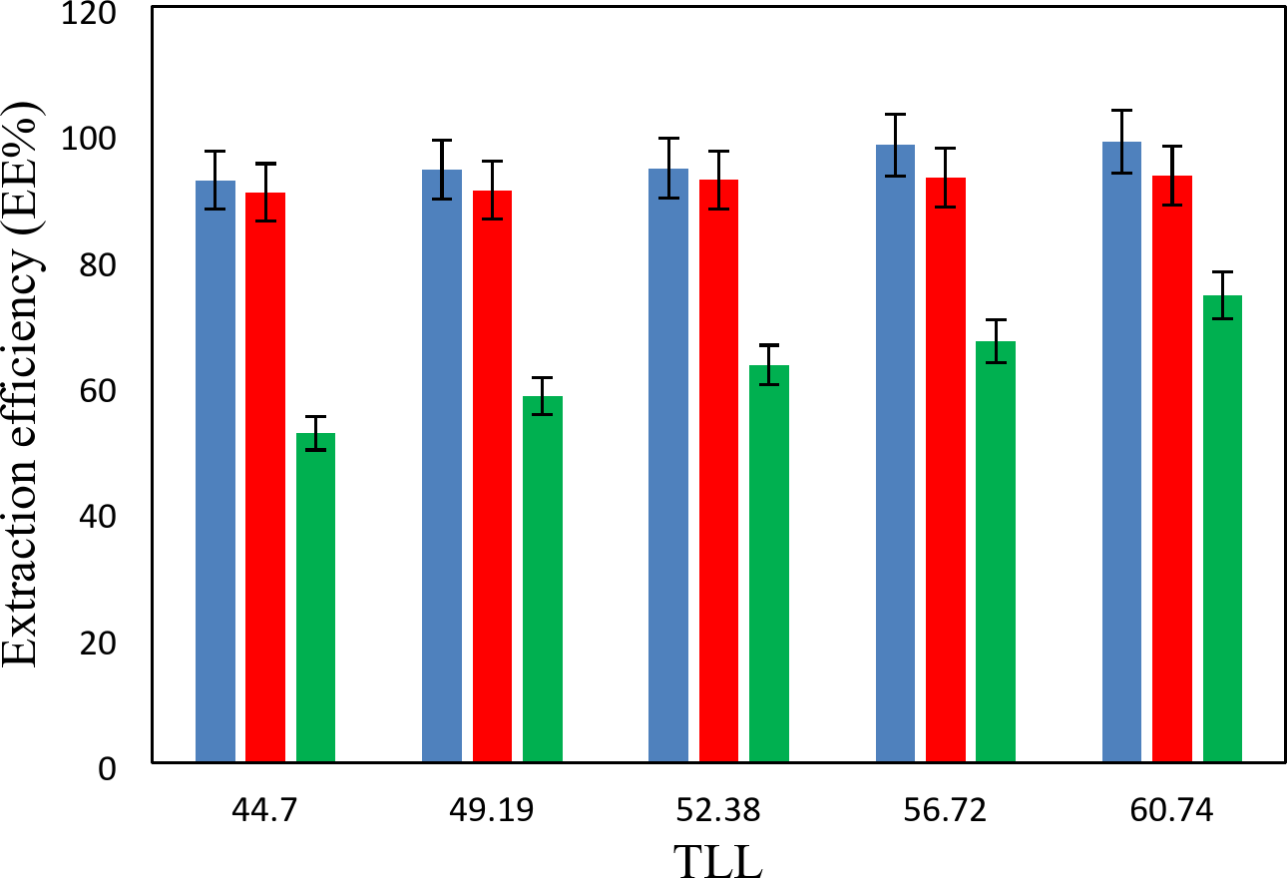
Partition coefficient, *K*, in function of the *TLL* for salicylic acid at *T* = 298.15 K: * Red square*, {betaine + K_3_PO_4_ + H_2_O}; * Blue square*, {betaine + K_2_HPO_4_ + H2O}; * Orange square*, {betaine + PEGDME_250_ + H_2_O}.

**Fig. 10:**
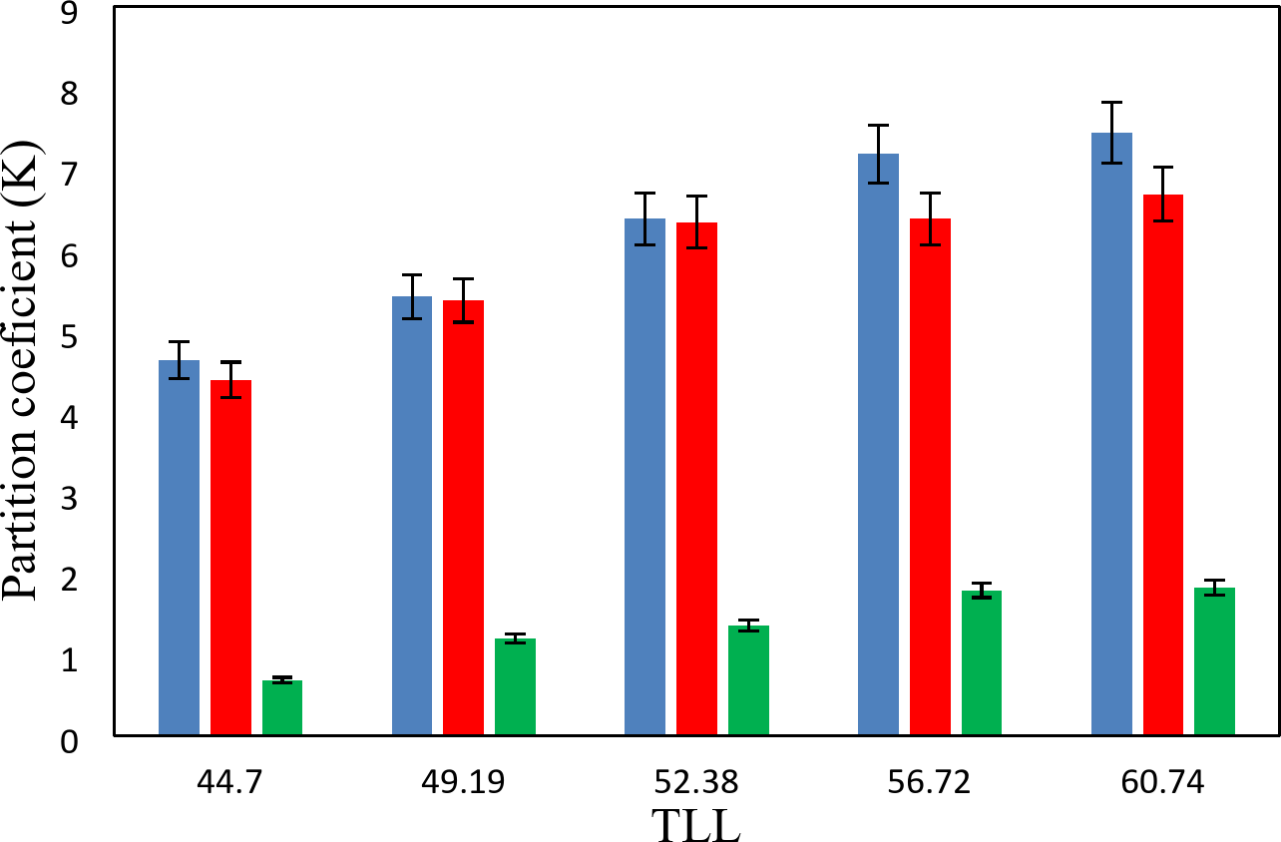
Partition coefficient, *K*, in function of the *TLL* for acetaminophen at *T* = 298.15 K: * Red square*, {betaine + K_3_PO_4_ + H_2_O}; * Blue square*, {betaine + K_2_HPO_4_ + H2O}; * Orange square*, {betaine + PEGDME_250_ + H_2_O}.

**Fig. 11:**
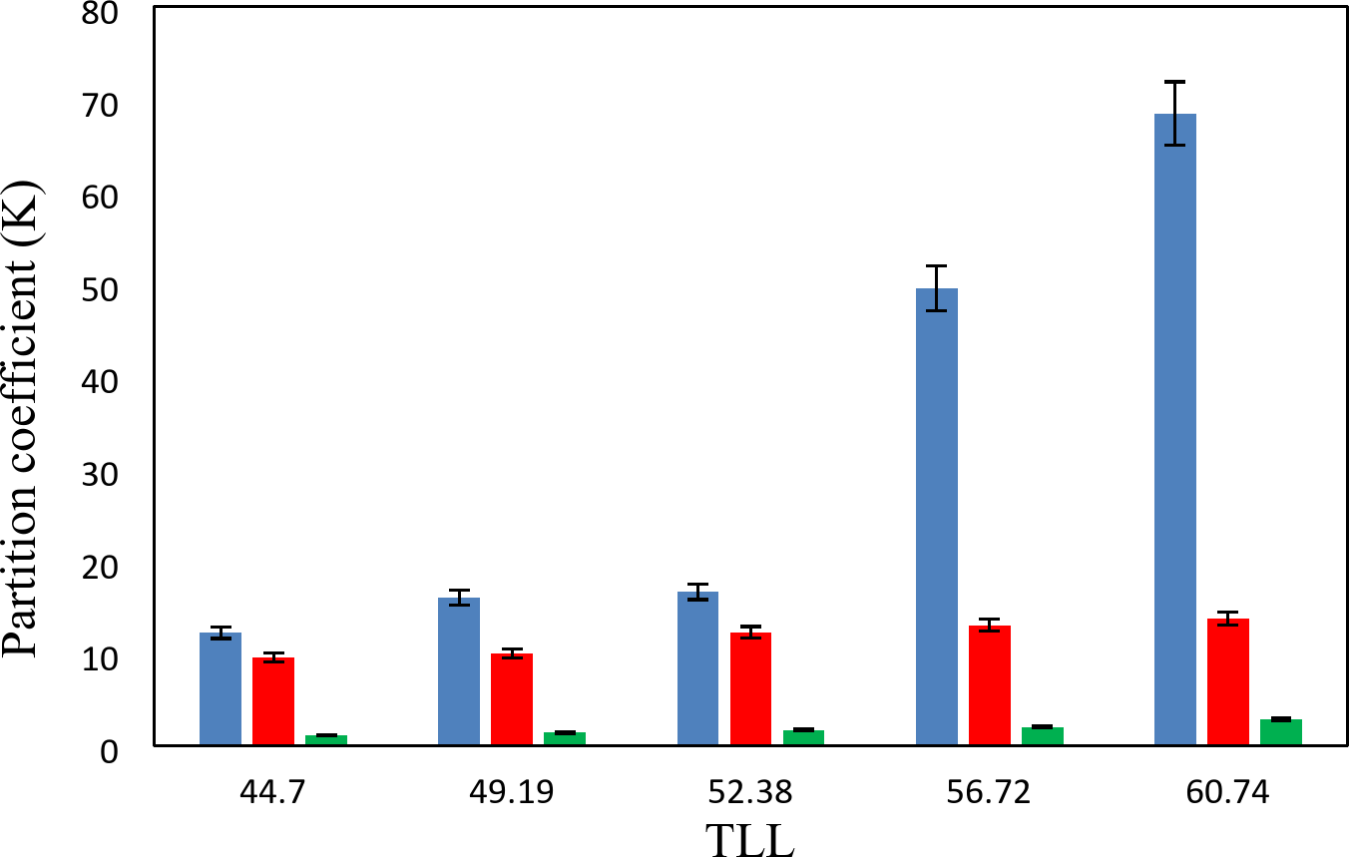
Partition coefficient, *K*, in function of the *TLL* for ceftriaxone at *T* = 298.15 K: * Red square*, {betaine + K_3_PO_4_ + H_2_O}; * Blue square*, {betaine + K_2_HPO_4_ + H2O}; * Orange square*, {betaine + PEGDME_250_ + H_2_O}.

The experimental partition coefficients, *K*, and the percentage extraction efficiencies, *EE*%, for the drugs under investigation, namely salicylic acid, acetaminophen and ceftriaxone are presented in Tables 13, 14 and 15. The obtained *EE*% and *K* values are also plotted against tie-line lengths for the studied drugs in Figs. 6, 7, 8, 9, 10 and 11, respectively. From variation of these *K* values, it is clear that the observed trend with all the three ATPSs used is consistently as follows: *K* (salicylic acid) > *K* (acetaminophen) > *K* (ceftriaxone). The data in Tables 13, 14 and 15 evidently indicates that the drugs predominantly partition into the more hydrophobic phase, which is the betaine-rich top phase. Also, close examination of the *K* or *EE*% values of Tables 13, 14 and 15 imply that the ATPS composed of betaine and K_3_PO_4_ is excellent in partitioning of the investigated drugs so that more than 98% of both the salicylic acid and acetaminophen can be extracted into top betaine-rich phase. This is because of the larger area of the two-phase region for the ATPS composed of betaine and K_3_PO_4_ hydrophobic drugs tend to partition preferentially into the more hydrophobic phase of the system. A larger two-phase region facilitates the partitioning of hydrophobic drugs by making the hydrophobic phase more distinct and accommodating, and this effect is often enhanced by increased temperature and salting-out conditions as explained above about the Gibbs energy of hydration of studied salts, the salting-out effect of K_3_PO_4_ is higher than K_2_HPO_4_ and PEGDME_250_ and the partitioning behavior of studied drugs in this system is better than two other systems. Using this ATPS more than 88% of ceftriaxone can be extracted. Acceptable results were also obtained using ATPS composed of betaine and K_2_HPO_4_ in regard with extraction of all the studied drugs. However, a good performance of ATPS containing betaine and PEGDME_250_ is only observed for extraction of salicylic acid. The observed trend for the *K* values of drugs is in good agreement with their hydrophobicity values defined by the logarithm of octanol/water partition coefficient (log *K*_ow_); so that higher log *K*_ow_values show more hydrophobicity of solute and its lower tendency to water molecules^29^. The log *K*_ow_values of drugs are 2.34^35^, 0.46^5^, and − 1.7^36^ for salicylic acid, acetaminophen and ceftriaxone, respectively. From these data, it can be expected all studied drugs have a tendency to top the hydrophobic phase (betaine-rich phase for systems containing salts (K_3_PO_4_, K_2_HPO_4_) and polymer- rich phase for the system containing PEGDME). Therefore, the drug extraction efficiency is expected to be in the order: salicylic acid > acetaminophen > ceftriaxone which is indeed observed experimentally. This study and previous one^23^ indicate that the hydrophobicity of drugs can be a useful criteria in predicting drug partitioning between two-phases of an ATPS.

**Table 13:** Aqueous two-phase system salicylic acid extraction efficiency (*EE*%) and mass ratio of top and bottom phases (*V*_R_) for the {betaine + PEGDME_250_ or salts (K_3_PO_4_, K_2_HPO_4_) + H_2_O} ATPSs in the distribution of salicylic acid molecules at *T* = 298.15 K and *P* ≈ 85 kPa.

Overall composition / wt %		K	EE%	V_*R*_
✓ PEGDME				
19.3 21.6 23.9 26.2 28.5	45.129 45.129 45.129 45.129 45.129	4.308 4.485 5.566 6.102 6.305	81.16 81.77 84.77 85.92 86.31	0.895 0.871 1.063 0.804 1.053
✓ K_2_HPO_4_				
19.0 20.7 22.4 24.1 25.8	27.436 27.436 27.436 27.436 27.436	10.696 13.124 13.265 13.514 14.361	91.45 92.92 92.99 93.11 93.49	0.905 0.948 1.096 1.158 1.148
✓ K_3_PO_4_				
19.0 20.7 22.4 24.1 25.8	27.436 27.436 27.436 27.436 27.436	12.405 26.174 32.223 69.922 70.942	92.13 96.32 96.99 98.59 98.61	0.959 0.986 1.297 1.042 1.208

^*a*^Standard uncertainties for mass fraction, temperature and pressure are *u* (*w*i) = 0.002, *u* (*T*) = 0.05 K and *u* (*p*) = 0.5 kPa, respectively.

**Table 14:** Aqueous two-phase system acetaminophen extraction efficiency (%*E*_trp_) and mass ratio of top and bottom phases (*V*_R_) for the {betaine + PEGDME_250_ or salts (K_3_PO_4_, K_2_HPO_4_) + H_2_O} ATPSs in the distribution of acetaminophen molecules at *T* = 298.15 K and *P* ≈ 85 kPa.

Overall composition / wt %		K	EE%	V_*R*_
✓ PEGDME				
19.3 21.6 23.9 26.2 28.5	45.129 45.129 45.129 45.129 45.129	1.094 1.390 1.709 1.848 2.864	52.26 58.17 63.10 64.90 74.12	0.992 0.989 1.012 0.918 0.965
✓ K_2_HPO_4_				
19.0 20.7 22.4 24.1 25.8	27.436 27.436 27.436 27.436 27.436	9.571 9.941 12.280 13.045 13.749	90.54 90.86 92.40 92.88 93.22	0.929 1.010 1.001 1.001 0.994
✓ K_3_PO_4_				
19.0 20.7 22.4 24.1 25.8	27.436 27.436 27.436 27.436 27.436	12.210 16.007 16.637 49.505 68.444	92.43 94.12 94.33 98.02 98.56	0.997 0.979 1.021 0.999 1.034

^*a*^Standard uncertainties for mass fraction, temperature and pressure are *u* (*w*i) = 0.002, *u* (*T*) = 0.05 K and *u* (*p*) = 0.5 kPa, respectively.

**Table 15:** Aqueous two-phase system ceftriaxone extraction efficiency (%*E*_trp_) and mass ratio of top and bottom phases (*V*_R_) for the {betaine + PEGDME_250_ or salts (K_3_PO_4_, K_2_HPO_4_) + H_2_O} ATPSs in the distribution of ceftriaxone molecules at *T* = 298.15 K and *P* ≈ 85 kPa.

Overall composition / wt %		K	EE%	V_*R*_
✓ PEGDME				
19.3 21.6 23.9 26.2 28.5	45.129 45.129 45.129 45.129 45.129	0.683 1.197 1.360 1.793 1.827	52.26 54.49 57.62 64.19 64.63	0.995 0.891 1.012 1.087 0.989
✓ K_2_HPO_4_				
19.0 20.7 22.4 24.1 25.8	27.436 27.436 27.436 27.436 27.436	4.391 5.373 6.342 6.379 6.686	81.45 84.31 86.38 86.45 86.99	1.104 0.968 0.995 1.032 1.104
✓ K_3_PO_4_				
19.0 20.7 22.4 24.1 25.8	27.436 27.436 27.436 27.436 27.436	4.634 5.423 6.391 7.183 7.446	82.25 84.43 86.45 87.78 88.16	1.019 0.989 0.994 1.117 1.016

^*a*^Standard uncertainties for mass fraction, temperature and pressure are *u* (*w*i) = 0.002, *u* (*T*) = 0.05 K and *u* (*p*) = 0.5 kPa, respectively.

### The effect of PH on the partitioning of drugs

Potassium phosphate can affect the pH of water when dissolved. K_3_PO_4_ and K_2_HPO_4_ are a salt of phosphorous acid (H_3_PO_4_), but in its dissociated form, potassium phosphate acts more like a base rather than an acid. When it dissolves in water, it dissociates into potassium (K⁺) ions and phosphate ions (PO_4_³⁻). Phosphate ions (PO_4_³⁻) can react with water molecules to form hydroxide ions (OH⁻) through hydrolysis. This reaction increases the concentration of OH⁻ in the solution, making the water more basic. As a result of the increased OH⁻ concentration, the pH of the water rises. This means that K_3_PO_4_ and K_2_HPO_4_will raise the pH of water, making it more alkaline^37,38^. As can be understood from Tables 13, 14 and 15, all three drugs have acidic groups and are a little soluble in the salt-rich phase.

#### Interpretation of the results

The phase diagrams of the studied systems provide crucial information about the conditions under which single-phase and two-phase regions exist. The binodal curves, obtained for the ternary systems {betaine + PEGDME250 + H₂O}, {betaine + K₃PO₄ + H₂O}, and {betaine + K₂HPO₄ + H₂O} at different temperatures, illustrate the extent of the two-phase region. The salting-out effect, which is more pronounced in systems containing salts than in those with the polymer PEGDME250, governs the formation of the two-phase region. This can be attributed to the hydration energy (*ΔG*_*hyd*_) of the anions involved. The highly negative *ΔG*_*hyd*_ of (PO_4_)^3−^ results in stronger salting-out effects, stabilizing the two-phase region and enhancing its applicability for drug partitioning.

Increasing temperature leads to an expansion of the two-phase region for all systems. For the polymer-based system, this is due to the disruption of hydrogen bonds between PEGDME250 and water, increasing the polymer’s hydrophobicity and facilitating phase separation. For salt-based systems, higher temperatures enhance the salting-out effect by altering ion-water interactions, reducing the solubility of certain solutes, and promoting phase separation. However, the temperature effect is moderate and system-specific.

The experimental binodal data were successfully correlated using three equations (Merchuk, Zafarani-Moattar and Nemati, and modified effective excluded volume theory). Among these, the modified effective excluded volume theory (Eq. 8) demonstrated superior performance with the smallest standard deviations, indicating its robustness in representing binodal data.

The plait point, representing the critical concentration where two phases become identical, was estimated using extrapolation methods. The tie-line data, essential for understanding phase equilibrium, were correlated using Othmer-Tobias, Bancraft, and Setschenow-type equations. The Setschenow equation showed the best performance, with parameters like *k*_*s*_ and k_c_ reflecting the salting-out ability and the system’s ability to partition components effectively. The higher *k*_*s*_ value for K_3_PO_4_ compared to K_2_HPO_4_ aligns with its stronger salting-out effect and greater phase separation efficiency.

The partitioning behavior of drugs (salicylic acid, acetaminophen, and ceftriaxone) in the studied systems is predominantly influenced by their hydrophobicity, as indicated by their log *K*_*ow*_ values. Drugs with higher hydrophobicity (e.g., salicylic acid) partition more effectively into the hydrophobic phase (betaine-rich phase for salt systems and polymer-rich phase for PEGDME systems). The larger two-phase region in the K_3_PO_4_-based system facilitates better partitioning and extraction efficiency, with salicylic acid and acetaminophen achieving over 98% extraction. The observed trend in drug partitioning (salicylic acid > acetaminophen > ceftriaxone) is consistent with their hydrophobicity and their solubility in the salt-rich phase.

The dissolution of K_3_PO_4_ and K_2_HPO_4_ in water raises the pH, making the solution more alkaline. This influences the partitioning of drugs with acidic groups, as their solubility in the salt-rich phase is reduced due to deprotonation. Consequently, the drugs preferentially partition into the more hydrophobic top phase.

### **Conclusion**

The three component aqueous systems containing betaine and PEGDME_250_, or phosphate salts (K_3_PO_4_ and K_2_HPO_4_) form two phase systems. From the obtained binodal curves at temperatures of 298.15, 308.15, and 318.15 K it was found that for the three {betaine + PEGDME_250_ + water}, {betaine + K_3_PO_4_ + water} and {betaine + K_2_HPO_4_} systems increasing the temperatures increases the two-phase region. The binodal data can be satisfactorily correlated to the three parameter Merchuk, Zafarani-Moattar et al. and the two-parameter modified equation derived from effective excluded volume theory; the performance of the two-parameter equation being excellent with the low standard deviations. Five tie-line data were obtained for each ATPS at 298.15 K and fitted to the Othmer-Tobias and Setschenow equations. We found that better results are obtained with Setschenow type equation, so that this equation can be satisfactorily used to reproduce the tie-line data for the all three investigated systems with relative standard deviations of 0.09, 0.03, and 0.04 for systems containing of PEGDME, K_3_PO_4_, and K_2_HPO_4_, respectively. The behavior of salting-out parameters estimated from Setschenow type equation is also consistent with the phase forming ability of these ATPSs.

In the next part of this research work the ability of these ATPSs were examined for three drug extractions. From the partitioning studying of the three drugs, acetaminophen, salicylic acid and ceftriaxone in these ATPSs and the calculation of partition coefficient and efficiency it was concluded that the drugs are mainly transferred to the top phase of these systems. With ATPS containing betaine and K_3_PO_4_ for both the salicylic acid and acetaminophen more than 98% and for ceftriaxone more than 88% can be extracted into top phase. Acceptable results were also obtained using ATPSs containing K_2_HPO_4_ or PEGDME_250_ in regard to extraction of all the studied drugs. Therefore, these betaine-based ATPSs represent a clean environment to green separation of the studied drugs. According to the trend of calculated extraction efficiencies the extent of extraction of drugs can be related to their hydrophobic properties.

## Electronic supplementary material

Below is the link to the electronic supplementary material.

Supplementary Material 1

## Data Availability

All data generated or analysed during this study are included in this article.
