# Supplementary material for: Phase equilibria and drug partitioning ability of betaine based aqueous two-phase systems
Source: Sci Rep. 2025 Feb 13;15:5395. doi: 10.1038/s41598-025-88326-4 (PMC11825666; doi:10.1038/s41598-025-88326-4)
Supplement: Supplementary file 1 — Supplementary Material 1 [file 41598_2025_88326_MOESM1_ESM.docx]

**Supporting Information**

**Phase equilibria and drug partitioning ability of betaine based aqueous two-phase systems**

**Mohammed Taghi Zafarani-Moattar,^*^ Hemayat Shekaari, Soheila Asadollahi**

*Department of Physical Chemistry, University of Tabriz, Tabriz, Iran*

*Corresponding author. [Tel: +98](Tel:+98) 4133393094 Fax: +98 41 33340191.

E-mail address: zafarani47@yahoo.com.

**Figure** **S1.** Effect of temperature on the binodal curve for {betaine + PEGDME_250_+H_2_O} system: (■), 298.15 K; (■), 308.15 K; (■), 318.15 K.

**Figure** **S2.** Effect of temperature on the binodal curve for {betaine + K_2_HPO_4_+H_2_O} system: (●), 298.15 K; (●), 308.15 K; (●), 318.15 K.

**Figure** **S3.** Effect of temperature on the binodal curve for {betaine + K_3_PO_4_+H_2_O} system: (▲), 298.15 K; (▲), 308.15 K; (▲), 318.15 K.

**Figure S4.** Binodal curve, tie-lines and plait point for the {betaine (1) + K_3_PO_4_ (2) + H_2_O (3)} system at *T*=298.15 K: (●) experimental binodal data, (▬▬) calculated binodal from Eq. (8), (---○---) calculated auxiliary curves, (▬▬▬) tie-lines data, (▬×▬) calculated from Eq. (12) and ■ plait point.

**Figure S5.** Binodal curve, tie-lines and plait point for the {betaine (1) + K_2_HPO_4_ (2) + H_2_O (3)} system at *T*=298.15 K: (●) experimental binodal data, (▬▬) calculated binodal from Eq. (8), (---○---) calculated auxiliary curves, (▬▬▬) tie-lines data, (▬×▬) calculated from Eq. (12) and ■ plait point.
